# Molecular Mechanisms of Epigenetic Variation in Plants

**DOI:** 10.3390/ijms13089900

**Published:** 2012-08-08

**Authors:** Ryo Fujimoto, Taku Sasaki, Ryo Ishikawa, Kenji Osabe, Takahiro Kawanabe, Elizabeth S. Dennis

**Affiliations:** 1 Graduate School of Science and Technology, Niigata University, Nishi-ku, Niigata 950-2181, Japan; 2 Gregor Mendel Institute of Molecular Plant Biology, Austrian Academy of Sciences, Dr. Bohrgasse 3, Vienna 1030, Austria; E-Mail: taku.sasaki@gmi.oeaw.ac.at; 3 Laboratory of Plant Breeding, Graduate School of Agricultural Science, Kobe University, Nada, Kobe 657-8510, Japan; E-Mail: r-ishika@port.kobe-u.ac.jp; 4 Cell and Developmental Biology, John Innes Centre, Norwich Research Park, Colney, Norwich NR4 7UH, UK; 5 Commonwealth Scientific and Industrial Research Organisation (CSIRO) Plant Industry, Canberra ACT 2601, Australia; E-Mails: kenji.osabe@csiro.au (K.O.); liz.dennis@csiro.au (E.S.D.); 6 Watanabe Seed Co., Ltd, Machiyashiki, Misato-cho, Miyagi 987-8607, Japan; E-Mail: tkawa@beach.ocn.ne.jp

**Keywords:** epigenetic, DNA methylation, metastable, natural variation

## Abstract

Natural variation is defined as the phenotypic variation caused by spontaneous mutations. In general, mutations are associated with changes of nucleotide sequence, and many mutations in genes that can cause changes in plant development have been identified. Epigenetic change, which does not involve alteration to the nucleotide sequence, can also cause changes in gene activity by changing the structure of chromatin through DNA methylation or histone modifications. Now there is evidence based on induced or spontaneous mutants that epigenetic changes can cause altering plant phenotypes. Epigenetic changes have occurred frequently in plants, and some are heritable or metastable causing variation in epigenetic status within or between species. Therefore, heritable epigenetic variation as well as genetic variation has the potential to drive natural variation.

## 1. Introduction

Variation in DNA sequence can cause variation in gene expression, which influences quantitative phenotypic variation in organisms and is an important factor in natural variation. Gene expression regulatory networks are comprised of *cis-*and *trans-*acting factors, and differences in gene expression are attributable to genetic variation. In eukaryotes, the genome is compacted into chromatin, and the chromatin structure plays an important role in gene expression: Gene expression can be controlled by changes in the structure of chromatin without changing the DNA sequence, and this phenomenon is termed “epigenetic” control. Recently, there have been many reports indicating that epigenetic change can cause phenotypic variation, and thus epigenetic change can be considered as an important factor in understanding phenotypic change. DNA methylation and histone modifications are well known epigenetic modifications. DNA methylation refers to an addition of a methyl group at the fifth carbon position of a cytosine ring, and in plants it is observed not only in the symmetric CG context but also in sequence contexts of CHG and CHH (where H is A, C, or T) [1–3]. DNA methylation is enriched in heterochromatic regions, such as in centromeric and pericentromeric regions, predominantly consisting of transposons [3–7]. Most transposons are immobile to protect genome integrity and are silenced via DNA methylation [3,8–12]. DNA methylation is also observed in euchromatic regions such as gene-coding regions (gene body methylation), and it is widely seen in eukaryotes [3,13,14].

Nucleosomes are formed by a histone octamer containing two of each of the core histones H2A, H2B, H3, and H4, and 147bp of DNA is wrapped around this core. The N-terminal regions of histone proteins are subject to various chemical modifications such as methylation or acetylation, and these histone modifications are associated with gene transcription [15,16]. In plants, DNA methylation, histone deacetylation and histone methylation in H3K9 (9th lysine of H3) and H3K27 are associated with gene repression, and DNA demethylation, histone acetylation and histone methylation in H3K4 and H3K36 are associated with gene activation [15,17]. Histone lysine residues are able to be mono-, di-, or tri-methylated and each methylation state is associated with different functions [15,17]. Epigenetic modifications play important roles in various aspects of the plant life cycle such as genome integrity, transgene silencing, nucleosome arrangement, nucleolar dominance, paramutation, flowering, and parent of origin-specific gene expression (imprinting) [15,18–21].

Genome-wide profiles of epigenetic information (the epigenome) are available in plants using new technologies such as tiling arrays or high-throughput next generation sequencing [15]. High-resolution maps of epigenetic features have been obtained from bisulfite sequencing (bisulfite converted DNA is directly sequenced) or a combination of chromatin immunoprecipitation (ChIP) technology and genomic tiling arrays (ChIP on chip) or ChIP and high-throughput sequencing (ChIP-seq) [15]. Using these technologies, effects of epigenetic modifications in mutants and variations of DNA methylation status between accessions in *Arabidopsis thaliana*, rice and maize have been shown at the whole genome level [22–26].

In general, heritable variation is a consequence of differences of nucleotide sequence. However, more studies are reporting heritable variation caused by epigenetic variation [27,28]. These epigenetic variations were categorized “obligatory”, “facilitated”, or “pure epialleles” by Richards [27]. “Obligatory” epigenetic variation is entirely dependent on DNA sequence changes, “facilitated” epigenetic variation is caused by stochastic variation in epigenetic status associated with a DNA sequence change, while “pure” epigenetic variation is generated stochastically and is completely independent of DNA sequence [27]. The “pure” epigenetic variations are subcategorized as stably or metastably inherited [29]. Sometimes these heritable epigenetic changes with or without genetic changes accompany phenotypic change, and there is evidence that spontaneous epigenetic changes generate new plant phenotypes in nature or in cultivars [30–32]. In addition, abnormalities in DNA hypo-methylated mutants have been characterized and some of them are due to the change of DNA methylation status without any difference in nucleotide sequence [33]. Increased knowledge about heritable epigenetic change associated with phenotypic variation suggests that heritable epigenetic changes may become a resource in plant breeding or play a role in plant adaptation [34].

In this review, we describe instances of naturally occurring epigenetic variants and how these can affect plant phenotype. We speculate on the possible causes and analyze the molecular basis of many of these variants and where possible, we elaborate on the resulting phenotypes. Most of our examples are from *A. thaliana,* as its genomics resources are most advanced. We conclude that epigenetic variation is widespread and contributes significantly to the generation of natural variation probably in most species, not just *A. thaliana*.

## 2. Epigenetic Variation Induced by Mutations of Genes Involved in Epigenetic Modification in *A. thaliana*

Epigenetic variation can arise in a number of ways. One way is through mutations in the genes responsible for maintaining epigenetic modifications such as DNA methylation. In *A. thaliana*, DNA methylation in the CG context is maintained by MET1 (METHYLTRANSFERASE 1), while non-CG contexts are maintained by DRM (DOMAINS REARRANGED METHYLTRANSFERASE) and CMT3 (CHROMOMETHYLASE 3) [3,35]. In addition to DNA methyltransferase, a chromatin remodeling factor| DDM1 (DECREASE IN DNA METHYLATION 1), histone methyltransferase| SUVH4/KYP (SU(VAR)3-9 HOMOLOG 4/KRYPTONITE) (hereafter KYP) and SUVH5/6, or SRA-domain methylcytosine-binding protein VIM1/2/3 (VARIANT IN METHYLATION 1/2/3) are also involved in the maintenance of DNA methylation [35]. The process of *de novo* DNA methylation is triggered by 24-nt siRNAs produced by the RNAi (RNA interference) pathway, termed RdDM (RNA-directed DNA methylation) [36]. Two plant specific RNA polymerases, Pol IV and Pol V, RDR2 (RNA-DEPENDENT RNA POLYMERASE 2), DCL3 (DICER-LIKE 3), and AGO4 (ARGONAUTE 4) proteins function in this RNAi pathway [36,37].

Plant developmental abnormalities have been detected in mutants with disturbed epigenetic modifications, some of which are heritable. An allele of a heritable variant, which is caused by a change in an epigenetic modification without a change in the DNA sequence, is termed an “epiallele”. In *A. thaliana*, *ddm1* hypo-methylated mutants showed only slight morphological changes in the early generations, but morphological abnormalities increased after repeated self-pollination over several generations [38,39]. Some developmental abnormalities are heritable and are not linked to the *DDM1* gene [40]. Some of these mutants are a consequence of the mobilization of transposons due to removal of DNA methylation from the transposon. Genes responsible for these abnormal phenotypes have been able to be identified by map-based cloning, because these phenotypes are heritable and indistinguishable from genetic mutations [33]. One of them is *clm* (*clam*), which showed a lack of elongation in shoots and petioles. This *clm* mutant is caused by an insertion of a *CACTA1* transposon in the *DWF4* gene, which encodes 22-α-hydroxylase in the brassinosteroid biosynthetic pathway. In wild type, *CACTA1* is silent, but it can transpose in *ddm1* [8]. This transposition has also been observed in *met1 cmt3* double mutants, indicating that DNA methylation is important for the silencing of *CACTA1* [41]. Another mutant, *wvs* (*wavy-sepal*), is also caused by an insertion of a transposon into the *FASCIATA1* gene. This transposon is a member of LTR (Long-terminal repeat) retrotransposon class, *AtGP3-1. AtGP3-1* is silent in wild type, but it can transpose in *ddm1* [11]. *clm* and *wvs* are genetic mutants caused by epigenetic changes. Another transposon is mobilized in the hypo-methylated mutants, *ddm1* or *met1*, and has the potential to generate a new genic mutant [11,42].

**Figure 1:**
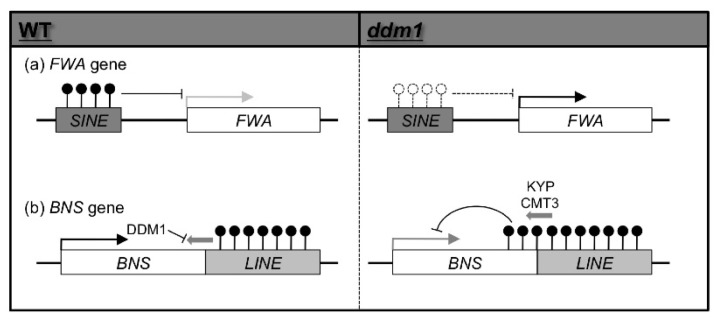
Epialleles in the *ddm1* mutant. (**a**) In WT (wild type) plants, expression of the *FWA* gene is repressed by DNA methylation of a promoter region-harboring short interspersed nuclear element (SINE) (left). In *ddm1* mutants, decreased DNA methylation in the SINE element induces ectopic expression of the *FWA* gene (right); (**b**) *BONSAI* (*BNS)* gene is flanked by LINE sequences, which are hyper-methylated, in tail-to-tail manner. In *ddm1* mutants, DNA methylation spreads into the *BNS* gene from the LINE sequence in a CMT3-KYP dependent manner, and stochastically induces silencing of the *BNS* gene.

Other mutants can be caused directly by changes in DNA methylation affecting transcription of the gene. The late flowering mutant *fwa* (*FLOWERING WAGENINGEN*) caused by ectopic expression of the *FWA* gene, encodes a homeodomain-containing transcription factor. In wild type, the promoter region of *FWA* is DNA methylated and *FWA* is not expressed in vegetative tissues, this DNA methylation is removed in the *ddm1* mutant and *FWA* is expressed in vegetative tissues (Figure 1) and causes late flowering [43]. This late flowering phenotype is also observed in the *met1* mutant [44,45], but not in the *drm1 drm2 cmt3* triple mutant [46], suggesting that silencing of *FWA* is mainly dependent on CG methylation. The DNA hypo-methylation in the promoter region of *FWA* and the late flowering phenotype are stable in the normal *DDM1* background, indicating that *fwa* is a gain of function epigenetic mutant.

Another mutant phenotype seen in the *ddm1* background, change of plant structure (short and compact inflorescence with reduced plant height), is *bns* (*BONSAI*), which is unstably inherited in the presence of the *DDM1* gene. The *BNS* gene encodes a protein with similarity to the mammalian cell cycle regulator Swm1/Apc13. In wild type, the *BNS* gene is normally expressed and not methylated (Figure 1). However, in a self-pollinated *ddm1* mutant, the *BNS* gene is methylated and stochastically silenced (Figure 1), indicating that *bns* is a loss of function epigenetic mutant. The *BNS* gene is flanked by a LINE (Long interspersed repeated element) sequence in a tail-to-tail orientation, and in the *ddm1* mutant DNA methylation in the *BNS* coding region spreads from the LINE (Figure 1). In *ddm1*, the DNA methylation level in *BNS* gradually increases over generations and a phenotype develops. There are two types (with or without LINE sequence) of variation in the *BNS* gene among 96 accessions of *A. thaliana*, 70 of these 96 accessions have LINE sequences at the *BNS* locus. Cvi that lacks the LINE sequence does not show DNA methylation at the *BNS* locus even in a *ddm1* background, indicating that the LINE is essential for the spread of DNA methylation in the *ddm1* mutant background [47]. This shows there is ectopic local DNA hyper-methylation of a specific locus in the global DNA hypo-methylation mutant, *ddm1*. Although small RNAs corresponding to the *BNS* locus accumulate in the *ddm1* mutant, ectopic induction of *de novo* DNA methylation at the *BNS* locus in the *ddm1* background was independent of the RdDM pathway because mutations in RdDM components such as RDR2, DCL3, AGO4, PolIV, and PolV did not affect *ddm1*-induced DNA methylation at the *BNS* locus [48]. However, KYP and CMT3 were essential for this ectopic DNA hyper-methylation at the *BNS* locus. In addition, meDIP (methylated DNA immnoprecipitation)-chip analysis revealed that *BNS*-like loci were widespread within the *A. thaliana* genome, and that they are DNA hyper-methylated in the *ddm1* mutant background in a CMT3-KYP-dependent manner. Although CMT3 is known for the maintenance of DNA methylation in the CHG context, CMT3-KYP dependent alternative *de novo* DNA methylation was found in all three contexts [48].

The *met1* mutants in *A. thaliana* also show developmental abnormalities such as reduced apical dominance, alterations in flowering time, floral abnormalities, curled leaves, embryogenesis, and formation of viable seeds [44,45,49,50], some of which are inherited even when the wild type allele is present. Genome-wide inheritance of hypo-methylation status even in the presence of the *MET1* wild type locus has been observed in an F_8_ population derived from hybrids between *met1* and wild type [51]. The floral abnormalities in the *met1* mutant or *MET1* antisense lines are due to DNA hyper-methylation and silencing of *SUP* (*SUPERMAN*) and/or *AG* (*AGAMOUS*) [52,53]. DNA hyper-methylation occurs at CT-rich repeats in the promoter of *SUP* or in the promoter and second intron of *AG*. This shows global DNA hypo-methylation by the *met1* mutation, which causes local DNA hyper-methylation: Stochastic non-CG methylation has been observed in the *met1* mutant [54].

The *drm1*, *drm2* or *cmt3* single mutants did not show any apparent phenotypes, but *drm1 drm2 cmt3* triple mutants showed pleiotropic phenotypes including developmental retardation, reduced plant size, and partial sterility [46,55]. Unlike *ddm1* or *met1*, the *drm1 drm2 cmt3* phenotype is completely recessive: Pleiotropic phenotypes are not inherited independently of the *drm* and *cmt3* mutations [55]. The misexpression of *SDC* (*Suppressor of drm1 drm2 cmt3*) was observed in the *drm1 drm2 cmt3* triple mutant, and it is sufficient for pleiotropic phenotypes in *drm1 drm2 cmt3* triple mutants. The promoter region harboring tandem repeat regions is densely methylated in all contexts in wild type, but DNA methylation in the promoter region is eliminated in *drm1 drm2 cmt3* triple mutant. F_1_ progeny between *drm1 drm2 cmt3* triple mutant and wild type show reversion of developmental phenotypes and the promoter region of *SDC* becomes methylated and *SDC* expression is lost. siRNAs corresponding to tandem repeat regions are expressed in wild type leading to DNA methylation of the *SDC* promoter region dependent on the RdDM pathway. Taken together, the pleiotropic phenotypes in the *drm1 drm2 cmt3* triple mutant are due to *SDC* misexpression caused by the elimination of DNA methylation in its promoter region [56].

## 3. Natural Variation of Epigenetic Status

It is well known that DNA sequence polymorphisms at a single locus or multiple loci cause phenotypic variation, and that they are important sources of variation in plants during evolution. In addition to DNA sequence polymorphisms, epigenetic variation has the potential to contribute to the natural variation of plant traits. Epigenome analysis aids in explaining how natural epigenetic variation causes phenotypic differences in plants. The DNA methylation status at the whole genome level has been examined in several species [1,2,4–7,13,14]. Accessions of a species may have been sourced from different environments and different epigenetic modifications selected over time to ensure optimum adaptation to specific environments. Variation of DNA methylation between accessions in *A. thaliana* occurs with gene-body methylation being more variable than DNA methylation of transposable elements among 96 natural accessions [22,25,26,57,58]. Differentially methylated regions have been detected in a comparison of whole genome DNA methylation statuses between two lines of rice or maize [23,24]. In the case of maize, differentially DNA methylated regions were generally observed in intergenic regions. Stable inheritance of DNA methylation was exhibited using near-isogenic lines of maize, though *trans*-acting control of DNA methylation was detected at a few regions [24]. These differences in DNA methylation could have consequences for differential expressions of genes.

**Figure 2:**
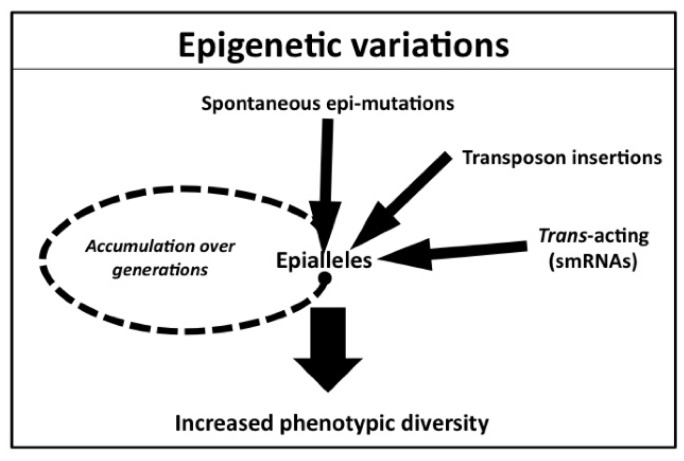
Factors that can lead to epigenetic variation in plants. Spontaneous epi-mutations, transposon insertions, and *trans*-acting (small RNAs) factors can contribute to the generation of epialleles. Epialleles can change gene expression and lead to phenotypic changes, and heritable epialleles can accumulate over generations and increase phenotypic diversity.

A comparison of DNA methylation statuses between parental lines and their progenies generated from single seed descent over 30 generations showed that larger regions of DNA methylation were stable and changes of DNA methylation accumulated through generations [59,60]. The rate of spontaneous changes of DNA methylation is higher than the rate of spontaneous genetic mutations [59–61], suggesting that sequence-independent epialleles play important roles in phenotypic diversity (Figure 2) [59,60]. To identify loci causing phenotypic variation, populations of epigenetic recombinant inbred lines (epi-RILs) between parents, which differed only in epigenetic marks, have been established in *A. thaliana*, and plant complex traits caused by epigenetic variation are observed [29,51,62]. In *A. thaliana*, two sets of epi-RILs were generated from *ddm1* or *met1* mutants that were crossed with wild type [29,48]. Stable inheritance of complex traits such as flowering time and plant height has been observed in these epi-RIL populations, providing important evidence that epigenetic variation can contribute to complex traits [29,51]. Heritable variation that was segregating in epi-RILs is similar to the phenotypic diversity observed in natural populations, suggesting that epigenetic variation in complex traits may drive some portion of natural variation (Figure 2) [63].

### 3.1. Spontaneous Epigenetic Mutants Occurring at Single Loci

Examples of spontaneous epi-mutants at single-loci, which influence plant traits, have been reported (Figure 2). Such epi-mutants are a change of flower structure from fundamental symmetry to radial symmetry in *Linaria vulgaris* (peloric) [30] and a *Cnr* (*colorless non-ripening*) mutant in tomato [31]. The peloric mutation is recessive and prevents expression of *Lcyc* (*Linaria cycloidea-like gene*) [30], and non-ripening of tomato fruit is due to the silencing of the *LeSPL-CNR,* which encodes an SBP-box (Squamosa promoter binding protein-like) transcription factor [31]. In these two cases, there is no sequence polymorphism between mutant and wild type, but high levels of DNA methylation of the causative genes were detected [30,31]. Occasionally some branches, which showed flowers near identical to wild type, were produced in the peloric plant population, and the flowers showed partial DNA demethylation in the *Lcyc* gene [30]. Similarly, the non-ripening phenotype in tomato is stable, but is reversible (showing normal ripening) at a low frequency [31]. In rice, the spontaneous dwarf mutant, *Epi-d1*, shows a metastable inheritance, and has been maintained for more than 90 years as breeding material like in the case of *LeSPL-CNR. Epi-d1* plants varied from dwarf to normal. The responsible gene, *D1* (*Dwarf1*), of *Epi-d1* encodes the α-subunit of a GTP-binding protein that is expressed differently between normal (active) and dwarf (inactive) plants, and this differential gene expression is not due to DNA sequence polymorphism. The silencing of the *D1* gene in *Epi-d1* is associated with H3K9 di-methylation in the genic region and DNA methylation in the *D1* promoter region. The promoter region harbors repeat regions, which show DNA methylation, and the repeat region is required for dwarf phenotypic metastability [32]. Tandem repeats are associated with paramutation at the *b1* locus of maize. Paramutation refers to the process where alleles interact in *trans* to establish meiotically heritable expression states [19], but *Epi-d1* did not show a paramutation-like phenotype [32]. These three examples reveal that spontaneous epigenetic changes can be metastably heritable for hundreds of years in nature or during domestication.

### 3.2. Transposon Insertion Can Generate Epigenetic Alleles

Transposon insertion in a coding region normally abolishes protein function, and there are some reports of insertion of transposons in a flanking region or intron of protein-coding genes, which can change the expression level of nearby genes [64–69]. Sometimes genetic variation such as transposon insertion drives spontaneous epialleles (Figure 2). Two cases in melon and *A. thaliana* showed that transposon insertion causes phenotypic change through the heritable epialleles.

Uni-sexual females (gynoecy) arise in melon by the action of a recessive *g* allele, which leads to a transition from male to female flowers. A 1.4 kb region was mapped at the *g* locus, which harbors a DNA transposon of the hAT family, termed *Gyno-hAT*. The insertion of *Gyno-hAT* downstream of *CmWIP1*, which encodes a C2H2 zinc-finger transcription factor of the WIP protein subfamily, induces DNA methylation in its promoter region, suggesting that DNA methylation caused by *Gyno-hAT* insertion suppresses *CmWIP* expression [70].

*A. thaliana* accessions can be categorized into early- and late-flowering, which is largely dependent on the allelic variation at two loci, *FRI* (*FRIGIDA*) and *FLC* (*FLOWERING LOCUS C*). Landsberg *erecta* (L*er*) accession is early flowering and shows low-level *FLC* expression [71,72]. The L*er FLC* allele (*FLC*-L*er*) has a non-autonomous *Mutator*-like transposable element insertion in the first intron, which may cause low-level *FLC* expression [71]. siRNAs corresponding to the inserted transposable element (TE) sequence accumulate, and HEN1 (HUA ENHANCER 1), SDE4 (SILENCING MOVEMENT DEFICIENT 2)/NRPD1 (Nuclear RNA polymerase D1A), and AGO4 are involved in this accumulation. High-level *FLC* expression with a late-flowering phenotype was observed in the *hen1-1* mutant, but *FLC* expression level or flowering time did not change in *ago4-1*. The TE in *FLC*-L*er* is DNA methylated, but surrounding regions were not. This DNA methylation of the TE was reduced in the *hen1-1* and *ago4-1* mutants, indicating that DNA methylation of the TE is not associated with *FLC* expression. However H3K9 di-methylation was detected in L*er* or *ago4-1*, but not in *hen1-1*, indicating that the level of H3K9 di-methylation inversely correlated with the level of *FLC* expression. This suggests that TE in *FLC*-L*er* results in low level of *FLC* expression through H3K9 di-methylation triggered by siRNA [73].

These two examples suggest that transposable insertion can drive the generation of new epialleles via changing the epigenetic modifications of nearby genes. In the epiallele, *bsn*, caused by hyper-methylation, DNA methylation in the *BNS* locus is dependent on the existence of a LINE transposable element (Chapter 2) [47]. Transposon insertion sites, number of transposons, and activity of transposons vary among accession of *A. thaliana* [74] and between *A. thaliana* and the related species, *Arabidopsis lyrata* [75,76], suggesting that distribution of transposable elements may drive natural variation via epigenetic changes in the nearby genes.

### 3.3. Trans-Acting Epigenetic Modifications

In addition to transposable element insertion, structural differences such as tandem repeats between accessions may trigger *trans*-acting DNA methylation and silencing through small RNAs (Figure 2). One example of *trans*-acting DNA methylation is the *PAI* (*Phosphoribosylanthranilate isomerase*) gene, which is involved in catalyzing the third step of the tryptophan biosynthetic pathway. The majority of *A. thaliana* accessions have three unlinked *PAI* genes, while in Ws and several other accessions, one of the *PAI* loci is rearranged as a tail-to-tail inverted repeat (IR) of two genes, *PAI1-PAI4* [77,78]. In Ws-type accessions, all four *PAI* genes are DNA methylated, while there is no DNA methylation in the three *PAI* genes in Col-type accessions [77,78]. The *pai* mutant in Ws, which lacks *PAI1-PAI4* IR, showed blue florescence under UV light and *PAI2* expression without DNA methylation [77]. The Col *PAI* genes were DNA methylated in the hybrid between Col and Ws [77], and transformation of Ws *PAI1-PAI4* IR into Col induced DNA hyper-methylation of *PAI* genes [79]. From these results, the IR structure triggers DNA methylation not only at *PAI1-PAI4* but also at the unlinked singlet genes *PAI2* and *PAI3*. A *cmt3* mutant or a *suvh4 suvh5 suvh6* triple mutant showed reduction of non-CG methylation at Ws *PAI* genes, and the *cmt3 met1* double mutant showed depletion of both CG and non-CG methylation [80–82]. Non-CG methylation at Ws *PAI* genes was reduced in the *dcl2 dcl3 dcl4* triple mutant, while DNA methylation did not change in *drm2* or *dcl3* mutants, indicating that a new pathway involving DCL-dependent small RNAs and the SUVH/CMT3 pathway but not involving the RdDM pathway controls DNA methylation at the Ws *PAI* genes [83]. RdDM independent but CMT3-KYP dependent *de novo* DNA methylation is observed in many loci in plants derived from *ddm1* [48], suggesting that the CMT3-KYP pathway is also involved in DNA methylation in *trans* by an uncharacterized mechanism.

DNA methylation in *trans* is also involved in plant reproduction. Sometimes hybrids between intra-specific accessions are unviable, which is known as hybrid incompatibility. The hybrid incompatibility caused by the genotypic combination of Col at the K4 locus and Sha (Shahdara) at the K5 locus is due to the lack of *AtFOLT* transcripts. In Col, *AtFOLT1* is expressed, but there is no *AtFOLT2* gene. In Sha, *AtFOLT2* is expressed, but *AtFOLT1* is not expressed. In Sha, lack of *AtFOLT1* expression was due to the high level of DNA methylation in its promoter region and there are siRNA transcripts corresponding to the promoter and first exon regions of *AtFOLT1*. The K4 locus in Sha comprises two additional rearranged truncated sequences homologous to parts of *AtFOLT2,* suggesting that siRNAs are produced from these rearranged gene copies and they can trigger *de novo* DNA methylation in *AtFOLT1* [84]. Further study will reveal which pathways, RdDM, CMT3-KYP, or others, are involved in *de novo* DNA methylation in *trans* via siRNAs.

DNA methylation in *trans* affects the expression not only of protein coding genes but also of transposable elements. The *MuK* (*Mu Killer*) locus dominantly silences an active *MuDR* [85,86]. As *MuK* results from an inverted duplication of a partially deleted autonomous *MuDR* element, it forms a perfect 2.4 kb hairpin RNA, which is processed into siRNAs [86]. *Muk* triggers DNA methylation of the terminal inverted repeats of *MuDR*. Once exposed to *MuK*, silencing of *MuDR* is heritable even in the absence of *MuK*, but *MuDR* elements can occasionally be reactivated with DNA demethylation when they are in a particular chromosomal position [85–87]. The *mop1* (*Mediator of paramutation 1*) mutant does not prevent the establishment of silencing of *MuDR* by *Muk*, but the NAP1 (Nucleosome assembly protein 1) knockdown mutant can. *MOP1* encodes a RNA-dependent RNA polymerase, which is an ortholog of *RDR2* in *A. thaliana,* and is involved in the production of 24-nt siRNAs, and NAP1 has been implicated as a histone chaperon [88,89]. The NAPs are required to establish a form of heritable silencing, perhaps by recruiting specific histone variants, but they are not required once the silencing state is established. By contrast, MOP1 is not required for the establishment of heritable silencing, but maintenance of *MuDR* silencing is assisted by MOP1 through the RdDM pathway [88].

**Figure 3:**
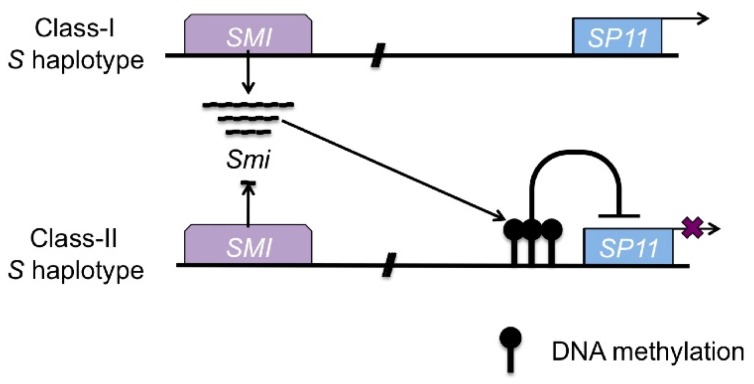
Dominance relationship in pollen. *Smi* derived from Class-I *S* locus can induce the *de novo* DNA methylation in the promoter region of Class-II *SP11*.

Another type of small RNAs, which can trigger *de novo* DNA methylation in *trans*, has been identified [90]. The dominance-relationship in the male determinant of self-incompatibility in Brassica is controlled by *de novo* DNA methylation in the promoter region of the recessive *S* determinant gene, *SP11/SCR* (*S locus protein 11/S locus cystein rich*), through small RNAs, *Smi* (*SP11 methylation inducer*). Self-incompatibility is controlled by one locus, the *S* locus, and the female-determinant gene of self-incompatibility, *SRK* (*S receptor kinase*), and *SP11/SCR* are located at the *S* locus [91,92]. As these two genes are inherited without recombination, they are called *S* haplotypes. As the self-incompatibility of Brassica is sporophytically controlled, there are dominant relationships between *S* haplotypes in the heterozygous plants on both the pollen and stigma side [92,93]. In Brassica, there are two types of *S* haplotypes, Class-I and Class-II, which are sequence based, and Class-I *S* haplotypes are dominant over Class-II *S* haplotypes in the Class-I/Class-II *S* heterozygote plants of pollen [92,93]. In Class-I/Class-II *S* heterozygotes, expression of Class-II *SP11/SCR* is suppressed and the promoter region of Class-II *SP11/SCR* is DNA methylated (Figure 3) [94]. The Class-I *S* haplotypes have the *SMI* (*SP11-methylation-inducing region*) located in the *S* locus, and its sequence has homology to the promoter region of Class-II *S* haplotypes (Figure 3). The 24nt-small RNAs, *Smi*, are expressed from *SMI*, and these small RNAs can trigger the *de novo* DNA methylation of the promoter region of Class-II *SP11/SCR* (Figure 3), indicating that Class-I derived *Smi* induces silencing of the recessive *SP11* allele by *trans*-acting *de novo* DNA methylation in the Class-I/Class-II *S* heterozygote plants [90]. Models of the molecular mechanism of *Smi* dependent *de novo* DNA methylation in *trans* have been suggested [95,96].

These four examples have revealed that genetic changes generating small RNAs can trigger *de novo* DNA methylation in *trans* during plant development. There are two types of *trans*-acting *de novo* DNA methylation, heritable as in paramutation or non-heritable, and several different molecular mechanisms induce *de novo* DNA methylation via small RNAs. These molecular mechanisms are generally plant specific and may be one factor generating natural variation.

## 4. Natural Variation of Imprinted *FWA* Genes in the Genus Arabidopsis

*FWA* is responsible for a late flowering phenotype in *A. thaliana* that is caused by the inhibition of FT function by protein-protein interaction between ectopically expressed FWA and FT [97,98]. *FWA* is expressed only in the central cell and endosperm in *A. thaliana* and reciprocal crosses between Col and L*er* have shown that only the maternal allele of *FWA* is expressed in the endosperm, indicating that *FWA* is an imprinted gene in *A. thaliana* [99]. The maternal allele is demethylated in the central cell by the demethylase, DME (DEMETER), which also acts on other imprinted genes in *A. thaliana* [99,100]. In vegetative tissues, DNA methylation of *FWA* occurs in the promoter region, which harbors two pairs of tandem repeats and a SINE (short interspersed nuclear element). This DNA methylation is reduced in the endosperm of *A. thaliana*, suggesting that methylation of this region participates in silencing of *FWA* [43,99]. Small RNAs are produced from the promoter region of *FWA*, suggesting that DNA methylation in this region is mediated by the RdDM pathway [101,102]. Indeed, DNA methylation of the promoter region of a “transgene” of *FWA* is dependent on the function of DRM2, RDR2, DCL3, and AGO4 [103]. Transformation of a double stranded RNA construct, which can cause *de novo* DNA methylation directed to a target region, into the *fwa* mutant has shown that DNA methylation in the region harboring the two pairs of tandem repeats and SINE region is sufficient for the silencing of *FWA* expression in vegetative tissues of *A. thaliana* [104].

**Figure 4:**
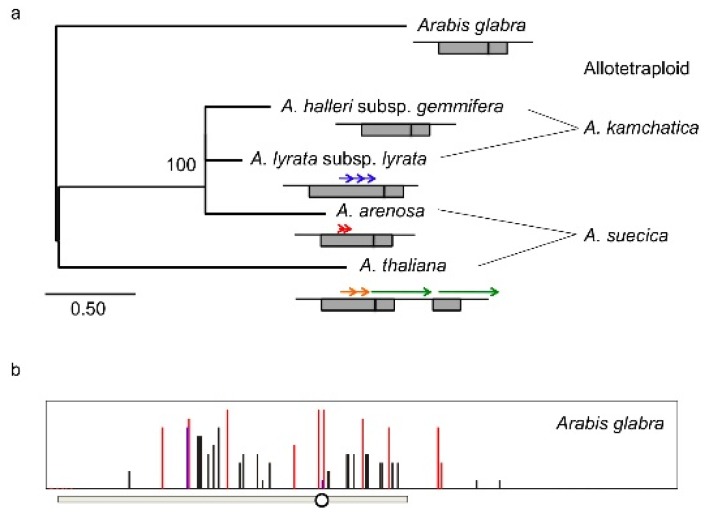
(**a**) Neighbor-joining tree of amino acid sequences of the FWA in the genus Arabidopsis. Bootstrap values with 1,000 replicates are indicated at the node of the neighbor-joining trees. *Arabis glabra* is used as out-group. Schematic views show the structure of the tandem repeats in the *FWA* promoter. Gray boxes reveal the SINE region, and vertical lines in the gray box show the transcription start site. Tandem repeats covering different regions are shown by different colors. *A. kamchatica* and *A. suecica* are allotetraploids between *A. halleri* and *A. lyrata* and between *A. thaliana* and *A. arenosa*, respectively; (**b**) Cytosine methylation status of the *FWA* promoter in *Arabis glabra*. Ten clones from bisulfite-treated templates were examined for each sample. Red, blue, and black bars represent methylation in CG, CHG, and asymmetric sites, respectively. Gray bars show the SINE-related sequences. The circle shows the transcription start site.

Using species related to *A. thaliana*, the structures that cause DNA methylation, imprinting, and vegetative silencing of *FWA* have been examined [105]. *FWA* genes are conserved in the genus in Arabidopsis, as there is high sequence homology not only in exon regions but also in the intron and promoter regions among species (Figure 4a). The SINE sequence is found in all species examined, *A. arenosa*, *A. halleri*, *A. lyrata*, *A. suecica* (allotetraploid between *A. thaliana* and *A. arenosa*) and *A. kamchatica* (allotetraploid between *A. halleri* and *A. lyrata*), suggesting that the SINE insertion is an ancient event (Figure 4a). In contrast, the structure of the tandem repeats is different among species: *A. halleri* and *A. halleri* allele of *A. kamchatica* have no tandem repeat in the SINE region, while *A. arenosa*, *A. lyrata*, *A. arenosa* and *A. thaliana* alleles of *A. suecica*, and the *A. lyrata* allele of *A. kamchatica* have tandem repeats like *A. thaliana* (Figure 4a). The sizes of the repeated and duplicated regions are different between species, suggesting that duplications occurred after speciation (Figure 4a) [105]. The ancient species, *Arabis glabra,* has a SINE region but no tandem repeat, supporting the hypothesis that the tandem repeat was not in the original structure (Figure 4). The *FWA* genes of *A. lyrata* and *A. halleri* show imprinted expression in immature seeds. DNA methylation of *FWA* in vegetative tissues in the SINE region is observed in all species. In *A. halleri* subsp. *gemmifera*, which lacks the tandem repeat structure, *FWA* shows imprinted expression, silencing in vegetative tissues, and DNA methylation in the SINE region, suggesting that the SINE sequence *per se* is important for epigenetic regulation of the *FWA* gene and *FWA* may have evolved silencing mechanism for transposable elements [101,105]. Transposable elements are extensively demethylated in endosperm, and the flanking regions of imprinted genes involving repetitive sequences are also demethylated, suggesting that imprinted genes evolved from targeted DNA methylation of transposable elements in *A. thaliana* [106,107].

**Figure 5:**
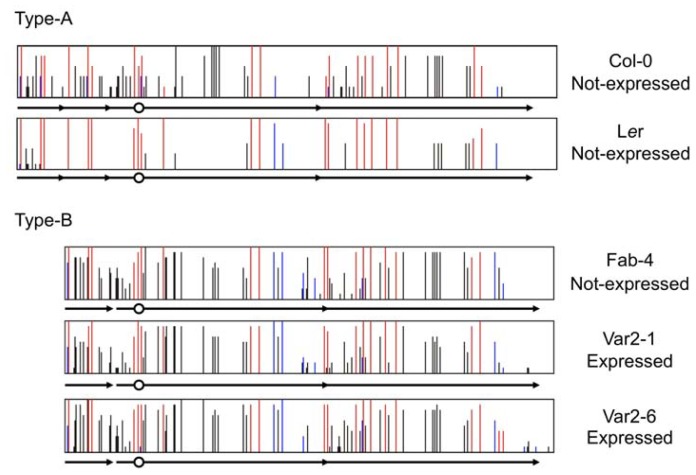
Cytosine methylation status of the *FWA* promoter in two types of accessions of *A. thaliana*. Type-A has short and large tandem repeats (shown by arrows), while Type-B has only large repeat. Ten clones from bisulfite-treated templates were examined for each sample. Red, blue, and black bars represent methylation in CG, CHG, and asymmetric sites, respectively. The circle shows the transcription start site. *FWA* is not expressed in vegetative tissues of Col, L*er*, and Fab-4, while being expressed in Var2-1 and Var2-6.

Vegetative silencing of *FWA* varies not only between species but also within species [105,108]. In *A. thaliana*, 93 out of 96 accessions have two pairs of tandem repeats (termed Type-A), and three have large tandem repeats but not short tandem repeats (termed Type-B). All 96 natural accessions have DNA methylation in the SINE region [22]. *FWA* is not expressed in all 21 accessions of Type-A that we selected randomly from 93 accessions, but two of three accessions of type-B, Fab-4, Var2-1, and Var2-6 showed a low level of *FWA* expression. However the DNA methylation level in the SINE region is almost the same among Type-B accessions (Figure 5). Though it is still unknown what the difference in the silencing stability among the three Type-B accessions is, two pairs of tandem repeats stabilize *FWA* silencing. Indeed, both large and small tandem repeats are involved in silencing *FWA* [105,108]. In *A. lyrata*, *FWA* is expressed in two strains of subsp. *lyrata* that has three tandem repeats and *FWA* is not expressed in subsp. *petraea* that has four tandem repeats. The *FWA* expression level tended to be inversely correlated with the DNA methylation level of the SINE in *A. lyrata* [105]. Another repeat in the subsp. *petraea* enlarged the DNA methylated region, suggesting that more tandem duplications might lead to greater stabilization of *FWA* silencing, similar to the indications from *A. thaliana*. In *A. halleri*, *FWA* was expressed in vegetative tissues of subsp. *halleri*, *tatlica*, and *ovirensis* and in several strains of subsp. *gemmifera*, while *FWA* was silenced in the majority of strains of subsp. *gemmifera*. One strain, IK, showed variation of *FWA* expression level among ten individual plants in spite of a perfect match of the promoter sequences, and there is a negative correlation between *FWA* expression level and DNA methylation level, especially with the non-CG methylation level in the region just upstream of the TSS (transcription start site) [105,108]. From these results, silencing of *FWA* is stable in Type-A of *A. thaliana* and *A. lyrata* subsp. *petraea*, but unstable in other species. This difference might be due to the number of tandem repeats, which can expand the DNA methylated region.

**Figure 6:**
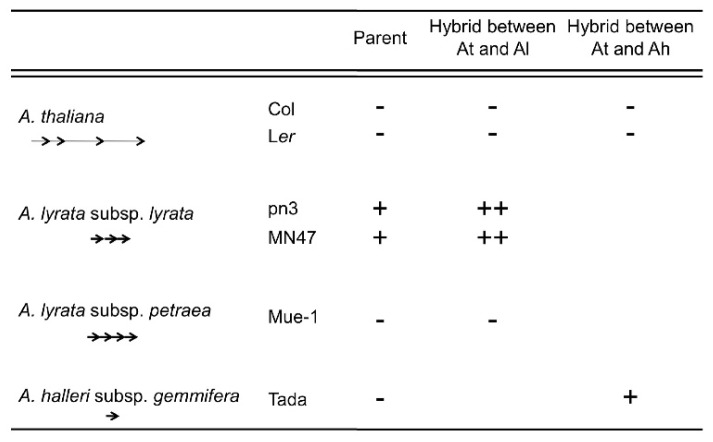
Summary of the vegetative *FWA* expression in inter-specific hybrids between *A. thaliana* and *A. lyrata* or between *A. thaliana* and *A. halleri*. Arrows show the tandem repeats in the *FWA* promoter. −; Absence of vegetative *FWA* expression, +; low level *FWA* expression in vegetative tissues, ++; More vegetative *FWA* expression. At; *A. thaliana*, Al; *A. lyrata*, Ah; *A. halleri*.

**Figure 7:**
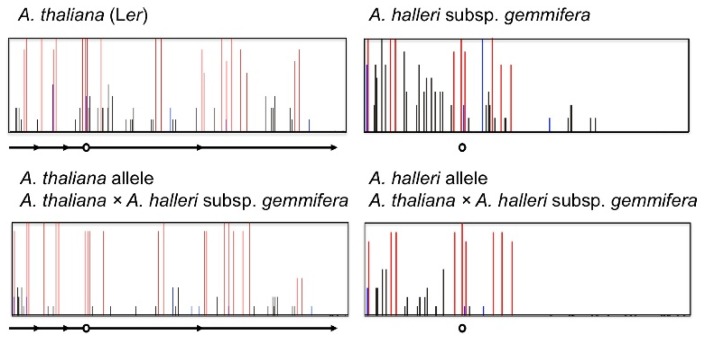
Cytosine methylation of the *FWA* promoter in *A. halleri* allele is reduced in the inter-specific hybrids between *A. thaliana* and *A. halleri* subsp. *gemmifera*, relative to direct parent. Ten clones from bisulfite-treated templates were examined for each sample. Red, blue, and black bars represent methylation in CG, CHG, and asymmetric sites, respectively. The circle and arrows show the transcription start site and tandem repeats, respectively.

**Figure 8:**
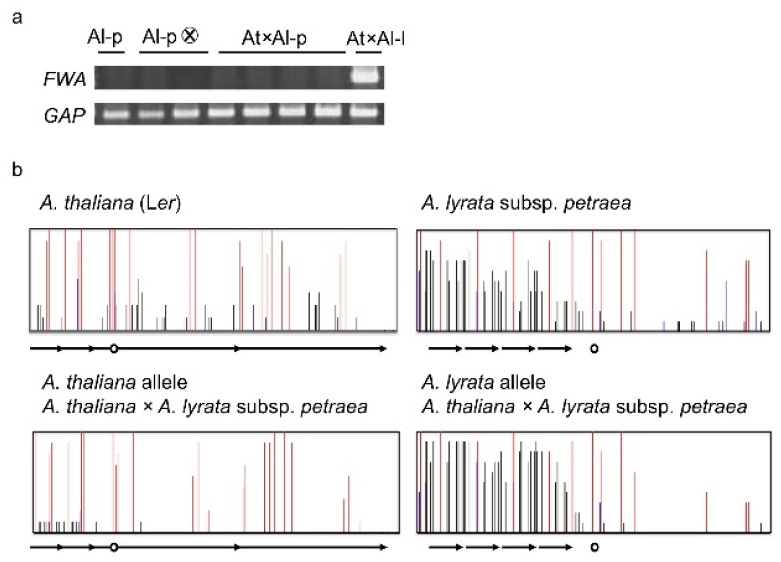
No alteration of vegetative *FWA* expression in the inter-specific hybrid between *A. thaliana* and *A. lyrata* subsp. *petraea*. (**a**) *FWA* transcripts in an inter-specific hybrid between *A. thaliana* and *A. lyrata* subsp. *lyrata* (Al-p). Al-l; *A. lyrata* subsp. *lyrata*; (**b**) Cytosine methylation of the *FWA* promoter in the inter-specific hybrid between *A. thaliana* and *A. lyrata* subsp. *petraea*. Ten clones from bisulfite-treated templates were examined for each sample. Red, blue, and black bars represent methylation in CG, CHG, and asymmetric sites, respectively. The circle and arrows show the transcription start site and tandem repeats, respectively.

The results from inter-specific hybridization support this suggestion. In the inter-specific hybrid between *A. thaliana* (Col or L*er*) and *A. lyrata* subsp*. lyrata* (pn3 or MN47), only the *A. lyrata* allele of *FWA* was expressed in vegetative tissues and this expression level was higher than the expression level of the parent *A. lyrata* subsp. *lyrata* (Figure 6). The DNA methylation level in the SINE region of the *A. lyrata* allele in the inter-specific hybrid was reduced in vegetative tissues [105]. In the inter-specific hybrid between *A. thaliana* (Col or L*er*) and *A. halleri* subsp. *gemmifera*, only the *A. halleri* allele of *FWA* was expressed in vegetative tissues in spite of no *FWA* expression in the parents (Figure 6). The DNA methylation level in the SINE region of the *A. halleri* allele of the inter-specific hybrids was also reduced in vegetative tissues, especially in the non-CG methylation of the region upstream of TSS (Figure 7). Though up-regulation of the *A. lyrata* or *A. halleri* allele in inter-specific hybrids was detected by RT-PCR, this up-regulation could not be detected by microarray analysis using ATH1 [109]. The *A. thaliana FWA* allele in two inter-specific hybrids was silenced and non-CG DNA methylation was slightly reduced in vegetative tissues (Figures 6 and 7). In the inter-specific hybrid between *A. thaliana* and *A. lyrata* subsp. *petraea*, there was no *FWA* expression in vegetative tissues as in their parents (Figure 6). The DNA methylation level in the *A. lyrata* allele of the inter-specific hybrid did not change (Figure 8). These results suggest that silencing of *FWA* might be affected by inter-specific hybridization, if the silencing level of the parent is unstable. These results also support the possibility of enhancement of silencing by tandem duplications.

From these results, two possibilities arise. (1) Tandem duplications stabilize *FWA* silencing, especially in Type-A of *A. thaliana*; (2) Non-CG DNA methylation in the region upstream of TSS is important for *FWA* silencing in the species related to *A. thaliana*. To confirm these possibilities, critical methylated residues controlling *FWA* silencing were examined using a double-stranded RNA to direct DNA methylation to target regions. In *A. thaliana*, DNA methylation in both short (region upstream of the TSS) and large tandem repeats (region downstream of the TSS) played a role in *FWA* silencing. In contrast, DNA methylation in the region upstream of the TSS played a role in *FWA* silencing in *A. lyrata* and *A. halleri*, but DNA methylation in the region downstream of the TSS was not sufficient for *FWA* silencing in *A. lyrata*. In *A. thaliana*, expression of small RNAs corresponding to the SINE region with two pairs of tandem repeats was confirmed, but few small RNAs were detected in *A. lyrata*, suggesting that DNA methylation in the SINE region is independent of the RdDM pathway in *A. lyrata*, unlike *A. thaliana* [101,102,104,108]. From these results, the critical methylated region for *FWA* silencing is different between *A. thaliana* and *A. lyrata*/*A. halleri*, and tandem duplications in *A. thaliana* enlarged the critical DNA methylated regions, which can stabilize the *FWA* silencing.

There is the question why the silencing mechanism is different between *A. thaliana* and species related to *A. thaliana*. This could be due to the ability of FWA to inhibit flowering in *A. thaliana* but not in *A. lyrata*. Over-expression of *FWA* from *A. lyrata* does not cause late flowering in an *A. thaliana* background, suggesting that *A. lyrata* FWA cannot inhibit FT function. Over-expression of both *A. thaliana FWA* and *A. thaliana FT* did not show any obvious developmental abnormality in flowers, but over-expression of both *A. thaliana FWA* and *A. lyrata FT* reveal occasional floral defects, which are due to misexpression of *AP1* (*APETALA1*) and *LFY* (*LEAFY*) [110]. *A. thaliana* shows amino acid changes in the C-terminal region of FWA close to the region important for binding of FT [98,108], suggesting that *A. thaliana* FWA might have gained the ability to interact with the FT protein after speciation. Thus ectopic FWA expression caused by DNA demethylation might be disadvantageous for both summer and winter annual natural accessions of *A. thaliana*, so *FWA* is stably silenced in *A. thaliana*. Tajima’s D test showed negative selection against mutations in the C/G site, suggesting that silencing of *FWA* mediated by DNA methylation plays an important role in adaptation of *A. thaliana* [105]. In *A. thaliana*, a more stable *FWA* silencing mechanism (spreading of critical methylated regions by tandem duplications) has been selected during the process of evolution. This can prevent late flowering caused by a newly generated FWA function involving spontaneous substitutions, which enable FWA to interact with FT and inhibit FT function.

## 5. Conclusions

Increasing numbers of epialleles are being reported in various species, and it is clear that epi-mutations can affect plant phenotypes. Some naturally occurring epialleles affect genes involved in plant fitness; some epialleles are stably or metastably inherited [34]. There are multiple causes of epi-mutations such as change of epigenetic status without genetic changes or via genetic changes such as transposon insertion or tandem repeat formations [34]. As plants are sessile organisms, they rely on adaptation mechanisms to withstand environmental stress. Phenotypic modifications by DNA sequence changes cannot respond quickly to environmental stresses. Metastable inheritance may be more useful in adaptation than genetic mutations because metastable epigenetic changes are more flexible and may contribute to phenotypic plasticity under environmental stress conditions [111,112]. Natural variation of epigenetic status has been found among accessions in several plant species, and this variation might be a consequence of the different growing condition in nature [15,20,112]. The higher epi-mutation rate has the potential to contribute to natural variation [59,60], and results using epi-RILs support the idea that complex epigenetic variations are one of the factors of natural variation [29,51]. More research focusing on naturally occurring epigenetic changes will increase our understanding of how epigenetic variation has contributed to natural variation.
